# Insight into the Mechanism of MXene Electrodes in Alkali Metal Batteries

**DOI:** 10.3390/nano16050330

**Published:** 2026-03-06

**Authors:** Sunaina Rafiq, Marco Agostini, Muhammad Abdullah Iqbal, Alessandra Gentili, Maria Assunta Navarra, Maria Grazia Betti, Carlo Mariani

**Affiliations:** 1 Dipartimento di Fisica, Sapienza Università di Roma, P.le Aldo Moro 2, 00185 Rome, Italy; maria.grazia.betti@roma1.infn.it (M.G.B.); carlo.mariani@uniroma1.it (C.M.); 2 Dipartimento di Chimica, Hydro-Eco Research Center, Sapienza Università di Roma, P.le Aldo Moro 2, 00185 Rome, Italy; marco.agostini@uniroma1.it (M.A.); alessandra.gentili@uniroma1.it (A.G.); mariassunta.navarra@uniroma1.it (M.A.N.); 3 Department of Physics, University of Kansas, Lawrence, KS 66045, USA; abdullah.iqbal@ku.edu

**Keywords:** MXene electrodes, alkali metal-ion batteries, electrochemical storage mechanism, electrochemical reversibility, intercalation and surface adsorption

## Abstract

The future growth of alkali metal-based batteries requires an understanding of how ion size affects the exchange mechanisms. In this work, we present a direct, comparative electrochemical study of MXene-based electrodes mechanism vs. lithium (Li^+^), sodium (Na^+^), and potassium (K^+^) ions using the same electrochemical conditions. This controlled method enables an extensive investigation of the size-dependent interactions between the MXene structure and alkali metal ions. X-ray photoelectron spectroscopy and Raman analysis of TMAOH-treated Ti_3_C_2_T_x_ MXene electrodes show that delamination and cycling alter vibrational modes and the surface chemistry. Voltage profile study reveals diverse storage behaviors: Li^+^ has a prominent intercalation plateau, Na^+^ shows intermediate properties, and K^+^ displays sloping profiles, indicating surface-dominated adsorption. The significant correlation between ionic radius and electrochemical reversibility is shown by long-term cycling data over 300 cycles, which show greater capacity retention and stability for Li^+^ and progressively lower performance for Na^+^ and K^+^. These findings provide new mechanistic insights into MXene–ion interactions and build the foundation for developing MXene-based materials for specific alkali-ion chemistries in next-generation energy storage devices.

## 1. Introduction

Energy storage systems are essential parts of modern technology, powering from portable devices and electric vehicles to stabilizing power grids that rely on intermittent renewable energy. Lithium-ion batteries (LIBs) are unique among these systems because of their high energy density, effectiveness, and small size [1,2]. LIBs have become the dominant rechargeable battery technology since Sony’s commercial launch in 1991, attributed to their high voltage, long cycle life, and environmental advantages. However, further developments are restricted by safety concerns, excessive costs [3], and upcoming theoretical performance limits [4,5,6,7,8,9]. The rise in the cost of lithium-ion batteries, due to lithium shortage and uneven resource distribution, has led to an effort for more affordable and effective alternatives [10,11]. Next-generation batteries that use more abundant metal ions are being actively researched to meet the increasing demands for energy [12]. In future, LIBs may eventually be replaced by sodium-ion batteries (SIBs) because of their accessibility and low cost [13,14], and for similar reasons also potassium-ion batteries (PIBs) are additionally gaining interest [15]. Additionally, multivalent-ion systems (e.g., Al^3+^, Mg^2+^, Ca^2+^) may transfer multiple electrons per ion, which results in increased energy density. Since sodium and potassium have similar chemical and physical properties to lithium and are found in far greater quantities in the Earth’s crust than lithium, these are currently hot research topics for SIBs and PIBs [16,17,18].

Most of the difficult challenges concern choosing the best electrolyte for each system and selecting host materials to serve as electrodes. Two-dimensional (2D) materials have great potential as host materials for metal ion batteries because of their unique structure, which allows for fast ion diffusion and provides more ion insertion channels available [19]. MXenes, belonging to a distinct class of 2D systems, have received a wide interest in recent years due to their unique structure and electronic properties. A wide variety of 2D transition metal carbides, nitrides, and carbonitrides called MXenes can be produced by treating the MAX phases with a wet hydrofluoric chemical. The general formula is M_n+1_X_n_T_x_ for MXene, derived from the M_n+1_AX_n_ phase, where “M” is an early transition metal, such as Ti or V, “A” is from elements such as Al or Si, “X” is nitrogen and/or carbon, and “n” is an integer (n = 1, 2, 3). The surface terminations (=O, OH, and F) that frequently result from the wet chemical etching methods used for producing MXenes from their MAX phase precursors are denoted by the term T_x_ [20]. They are projected to become new-style anode materials with outstanding conductivity, excellent cycle performance, and superior rate capability [21,22]. As previously stated, MXenes primarily contain the functional groups –OH, –O, and –F; these functional groups have an important effect on the electrochemical performance of MXenes as anode materials in rechargeable batteries. Several MXenes exhibit good ion transport properties, with low diffusion barriers for several ions such as Li^+^, Na^+^, K^+^, and Ca^2+^. Fan et al. [23] applied density functional theory to calculate the diffusion barriers of Li^+^, Na^+^, K^+^, and Ca^2+^ on a V_3_C_2_ monolayer. The results were 0.04 eV, 0.02 eV, 0.01 eV, and 0.04 eV, respectively, which were considerably less than those on other 2D materials (Li^+^ on graphene, 0.28 eV; Li^+^ on phosphorene, 0.13–0.76 eV) [24,25]. Notably, K^+^ exhibits the lowest diffusion resistance on V_3_C_2_, suggesting that PIBs using MXenes as the electrode material may have excellent rate performance and good quick charge/discharge capabilities. Due to these beneficial properties, MXenes are particularly attractive electrode materials for PIBs.

In this study, we present a systematic evaluation of MXene-based electrodes with lithium, sodium, and potassium ions under identical conditions to determine the effect of ion size on electrochemical performance. Distinct voltage profiles correspond to different storage methods, with lithium indicating a prominent intercalation plateau and potassium preferring surface adsorption. Long-term cycling over >300 cycles confirms the lithium superior capacity and stability, while potassium exhibits the lowest performance. The surface chemistry of the electrodes prior and after alkali metal cycling has been carefully investigated by X-ray photoelectron spectroscopy (XPS) and their vibrational/structural modification by Raman spectroscopy. The results obtained provide new insights on ion–MXene interactions and highlight the importance of ion size in the development of high-performance MXene-based energy storage systems.

## 2. Materials and Methods

### 2.1. Synthesis of Delaminated-MXene Nanosheets

**Figure 1:**
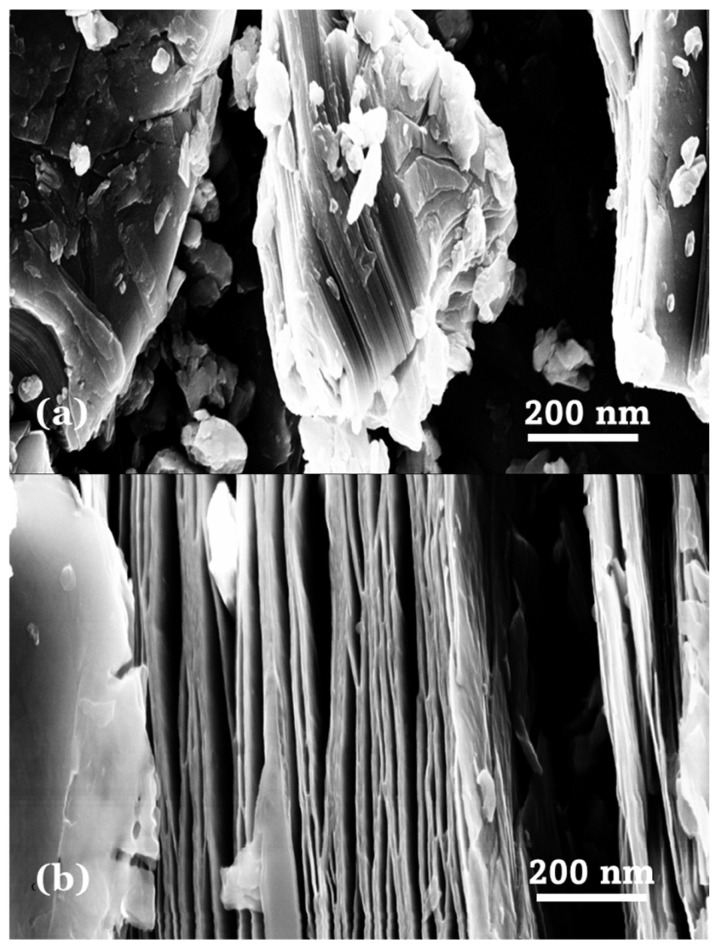
Representative SEM images of (**a**) MAX (Ti_3_AlC_2_) pristine powder and (**b**) multi-layer Ti_3_C_2_T_x_ powder after delaminating treatment with 49 wt.% HF.

The 2D MXene sheets preparation was performed by exposing 3 g of Ti_3_AlC_2_ powder (98 wt.% purity) to a 49 wt.% solution of HF (70 mL) in a Teflon bottle. The mixture was stirred constantly on a magnetic stir plate at room temperature (70 h). After the etching was done, the mixture was washed several times using deionized (DI) water. The slurry was then centrifugated repeatedly at 7000 rpm for 7 min until the pH of the suspension was stabilized at ∼6. The completely purified slurry was dried in a vacuum oven at 58 °C for 24 h to produce etched Ti_3_C_2_T_x_ MXene sheets. For organic base treatment, Tetramethylammonium hydroxide (TMAOH) was used to delaminate the Ti_3_C_2_T_x_ nanosheets. 100 mg of multi-layered Ti_3_C_2_T_x_ MXene powder was dissolved in 10 mL of DI water and 0.46 mL of TMAOH was put in the 20 mL of a glass vial. The vial was kept on a magnetic stir plate and was stirred for 14h at room temperature. Subsequently, the basic suspension was moved to centrifuging tubes and centrifuged twice for 10 min to lower the pH of ∼10 to ∼7. After 1 h more centrifugation, a clear colloidal MXene dispersion was acquired. The shaking then continued using a hand to delaminate the Ti_3_C_2_T_x_ nanosheets, thereafter the sediment was collected and dried at 120 °C in an oven [26]. The conversion of pristine Ti_3_AlC_2_ to Ti_3_C_2_T_x_ MXene, which is signified by a loose multi-layered structure, is evidence of the successful removal of the Al layers and thus the effectiveness of the etching protocol. Figure 1 illustrates SEM images of MAX (Ti_3_AlC_2_) pristine powder and multi-layer Ti_3_C_2_T_x_ powder after delaminating treatment with 49 wt.% HF. These images show the distinct transition from stacked multi-layered morphology to delaminated sheet-like structures.

### 2.2. Assembly of Coin Cell Batteries

In the manufacturing of battery electrodes, the slurry method was used to fabricate electrodes. The materials used included delaminated Ti_3_C_2_T_x_ MXene treated with TMAOH as the active material, Carbon Super P (Timcal, Bodio, Switzerland), Polyvinylidene fluoride (PVDF, Merck, Darmstadt, Germany), and N-Methyl-2-pyrrolidone (NMP, Merck) as the solvent.

The slurry mixture included 160 mg of active material, 20 mg of Carbon Super P, and 20 mg of PVDF were mixed in 2 mL of NMP. This mixture was stirred for 2 h, followed by manual milling. Then, using a doctor blade, the slurry was applied on copper foil at a thickness of 150-microns. Following the drying process, electrode disks with a diameter of 10 mm were cut, and the weight of the material was measured, resulting in an active material loading of about 2 mg/cm^2^.

For coin cell assembly (type 2032), the electrodes were transferred to a glove box (O_2_ and H_2_O < 1 ppm). MXene-based electrodes, a Whatman separator that had been soaked in electrolyte, and alkali metal disks (Li, Na, K) were used to make the cells. Electrochemical measurements were made using 1 M LiPF_6_, NaPF_6_ or KPF_6_ dissolved in 1:1 (*v*:*v*) EC/DMC solvent mixture. All the systems had the same salt concentration, the anion (PF_6_^−^), and the solvent system to provide a direct comparison to isolate ion-size effects. The EC/DMC electrolyte PF_6_-systems were chosen because their stability and SEI -forming behavior are well-documented benchmark systems. Current collectors were made of stainless-steel spacers, and a spring was placed to maintain a constant stack pressure and minimize contact resistance during cycling. Finally, the assembled cells’ voltage was tested using a galvanostatic cycling instrument, i.e., Maccor.

### 2.3. Characterizations Techniques

The XPS experiments were carried out in an ultra-high vacuum (UHV) chamber with a base pressure in the low 10^−10^ mbar range. An X-ray photon source (PSP TA10) with Mg-Kα photons (hv = 1253.6 eV) was used. A hemispherical VG Microtech Clam-2 electrostatic electron analyzer (VG Microtech Ltd., East Grinstead, UK) with an overall energy resolution better than 1 eV and constant pass energy mode set at 50 eV was used to measure the photoelectrons. The Au 4f_7/2_ core-level was set to 84.0 eV binding energy (BE) to calibrate the binding energy scale on a freshly sputtered gold foil in good electrical contact with the sample. Raman spectroscopic studies were performed using a Dilor LabRam micro-Raman spectrometer (Dilor S.A., Lille, France) with a Helium-Neon laser with a wavelength of 632.8 nm and a power of 4.7 mW with a grating of 1800 grooves/mm.

## 3. Results

### 3.1. X-Ray Photoelectron Spectroscopy Analysis

MXene (Ti_3_C_2_T_x_) was treated with TMAOH, which is a highly used method of delamination and an increase in accessible surface area; the mixture was then utilized in electrochemical studies. X-ray photoelectron spectroscopy was used to analyze the surface composition of the pristine delaminated Ti_3_C_2_T_x_ electrodes, and of the three recovered electrodes after the 100th cycle in Li, Na, and K cells. The recovered electrodes were exposed in an inert atmosphere of argon in the transfer to reduce oxidation and contamination of the electrodes during handling. XPS survey spectra in a wide BE range (shown in Figure S1 of the Supplementary Information), show the main core level peaks associated with the different elements. For the pristine delaminated sample, we can clearly identify the Ti 2p and C 1s core levels, along with the main Auger transitions, and a small oxygen contamination. For the samples used as electrodes after 100 cycles with Li, Na and K, we observe the alkali metal (AM) related core levels, and the C and O 1s core levels, while the Ti peaks are very faint, and the Na-cycled one also presents light Zn contamination, very likely deriving from the sample holder. There was no sputter etching in the X-ray photoelectron spectroscopy, since the Ar^+^ ion bombardment can alter the terminations of Ti_3_C_2_T_x_. Post-cycling contamination by solid electrolyte interphase (SEI) and electrolyte products is unavoidable; a more in-depth XPS study of uncontaminated MXene is presented in Ref. [26].

**Figure 2:**
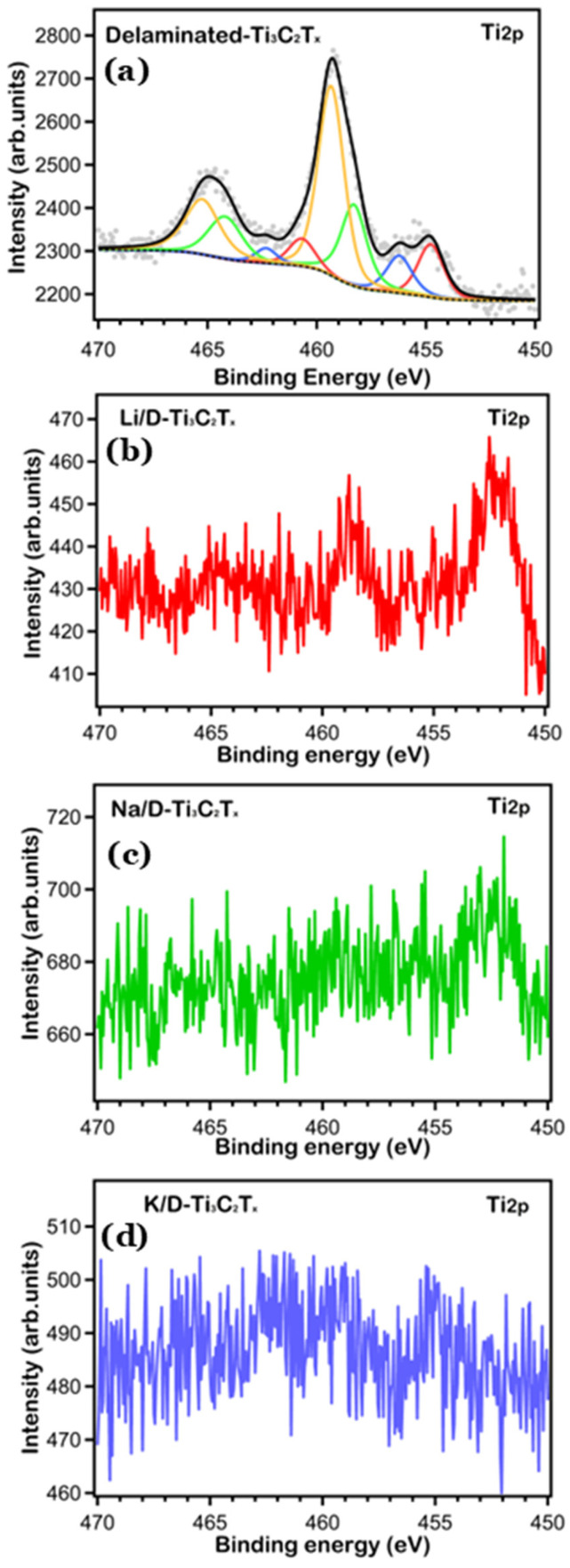
Ti 2p spectra of all samples: (**a**) pristine delaminated-Ti_3_C_2_T_x_; (**b**) Li-cycled D-Ti_3_C_2_T_x_; (**c**) Na-cycled D-Ti_3_C_2_T_x_; (**d**) K-cycled D-Ti_3_C_2_T_x_.

**Figure 3:**
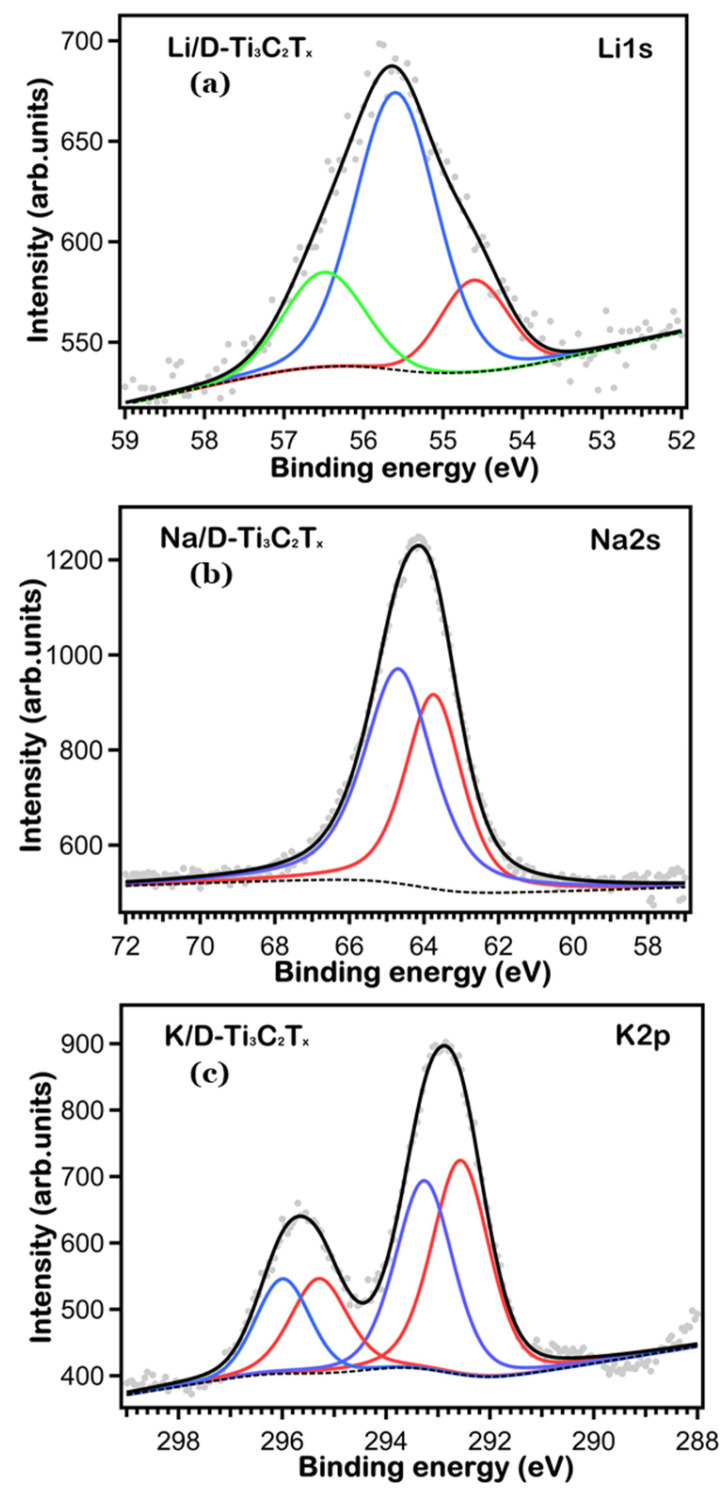
High-resolution XPS spectra of (**a**) Li 1s; (**b**) Na 2s and (**c**) K 2p core levels on the AM-cycled D-Ti_3_C_2_T_x_ electrodes.

**Figure 4:**
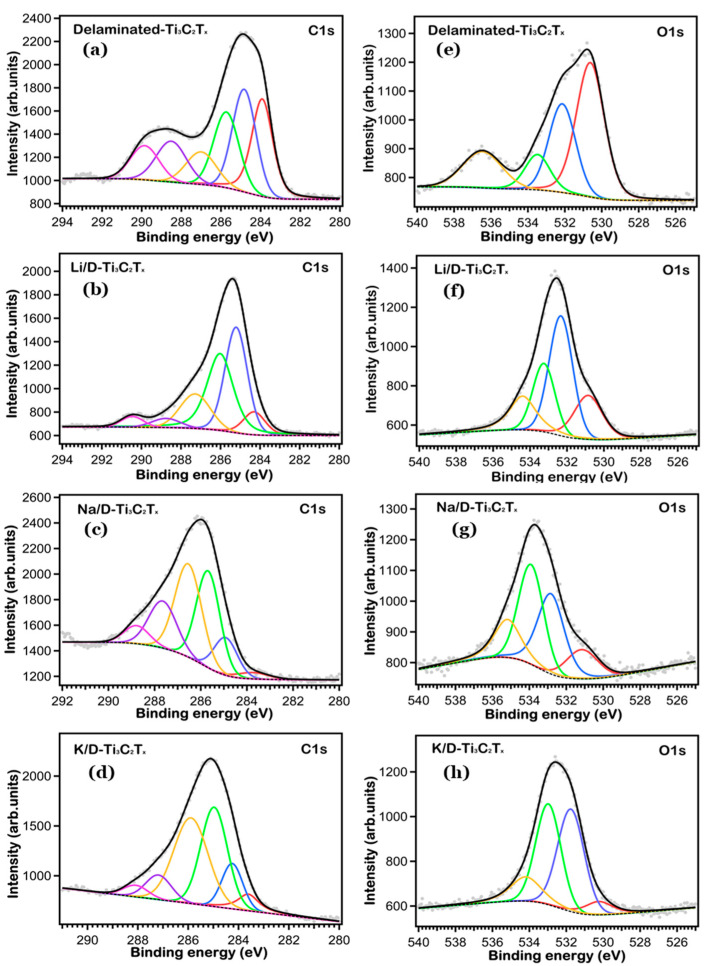
High-resolution spectra of the C 1s (**a**–**d**) and O 1s (**e**–**h**) of all the samples.

To get more quantitative information about the chemical state and surface compositions, we took XPS core level data with higher resolution and performed a fitting analysis. The high-resolution Ti 2p, Li 1s, Na 1s, K 2p, O 1s and C 1s core levels are shown in Figure 2, Figure 3 and Figure 4, respectively. We fitted the core level data to single out the peaks associated with the different chemically shifted components, by using pseudo-Voigt functions (Gaussian–Lorentzian curves, where the Gaussian part is associated with the overall experimental uncertainty and the Lorentzian one to the intrinsic excitation lifetime), after subtracting a Shirley background. The single fitting curves and the overall total fitting are superimposed to the experimental XPS data in Figure 2, Figure 3 and Figure 4, the fitting data (BE position, atomic % ratio, linewidth) are reported in Table 1, Table 2 and Table 3.

It is well known that after delamination with TMAOH, the surface of MXene (Ti_3_C_2_T_x_) exhibits a variety of surface terminations. This is reflected in the chemical shifted components of all the core levels due to various surface-groups attached to all the samples [27]. We noticed that the percentages we obtained for each of the measured elements changed after the cycling process, and they are not completely in line with the expected chemical composition of the pristine Delaminated-Ti_3_C_2_T_x_ spectrum. Since peak deconvolution is not a specificity of cycled MXene electrodes, the focus in the discussion presented below is placed on strong spectral patterns, i.e., positions of peaks and changes in their intensities, and then interpreted components fitted by assignments suggested by the literature. The Ti 2p spectra (Figure 2) present the typical spin orbital split (2p_3/2_ and 2p_1/2_) components and exhibit different Ti oxidation states. On the surface of the pristine D-Ti_3_C_2_T_x_, according to the fitting to our experimental data, we identify Ti 2p_3/2_ peaks at 454.7, 456.2, 458.3 and 459.3 eV B.E, which can be associated with TiC, Ti^+2^, Ti^+3^, and Ti^+4^, respectively, in agreement with the literature [28,29,30]. Through electrochemical cycling, it is observed that the Ti signal is highly attenuated which is attributed to the fact that the MXene surface is dominated by the presence of electrolyte-based species and it forms an SEI/interphase layer that prevents the display of the photoelectron signal of the underlying active material [31]. These findings support the Raman measurements discussed later, which suggest the presence of a Solid Electrolyte Interface (SEI) layer on the electrode surfaces.

As it concerns the core levels associated with the AMs, cycling of the electrodes leads to notable changes in both the binding energy and intensity of all fitted peaks in the Li, Na, and K spectra, as shown in Figure 3. Analysis of the fitted core levels indicates the formation of various interphases on the electrode surface due to the cycling process, and the identified changes in the positions of the peaks and relative intensities indicate the surface chemical environment transformation after cycling, which consequently validates the evidence of interphase formation in the literature.

Such shifts in the peaks can be caused by the alkali-metal coordination to the surface terminations of MXene and/or the interphase species formation containing alkali elements. Since, pristine Ti_3_C_2_T_x_ already has –O/–OH/–F terminations, the observed BE reduction after cycling is most likely the result of changes in their local chemical environment caused by electrolyte-derived surface species and alkali-metal intercalation/coordination [32].

More in detail, the fitting results of the Li 1s and Na 2s XPS data are reported in Table 1. The broad peak at 55.7 eV in Li 1s (Figure 3a) suggests the presence of three components at 54.6, 55.5 and 56.4 eV BE, associated with Li_2_O_2_/LiF, Li-O-H, and Li_2_-C-O_3_ [8,9]. In the case of Na 2s (Figure 3b), the broad structure at 64.2 eV indicates the presence of two peaks at 63.72 and 64.69 eV BE [33], due to Na^+^ ions in Na-F and bonded with oxygen (-O) in the cubic phase, respectively. In separate samples, the species of Li-F and Na-F are clearly observed, which suggests strong interactions of surface terminations and/or fluoride-incorporated interphase products exist on the MXene surface.

The K 2p core-level XPS profile is de-convolved into spin–orbit split doublets K 2p_3/2_ and 2p_1/2_ (with an area ratio of 2:1 and a BE difference of about 2.7 eV) [12]. The K 2p components suggest that the surface chemistry of potassium has changed after the cycling process (Figure 3c), this finding also suggests the existence of other K-containing surface species, as has been observed in oxide/carbonate-type products reported in cycled electrodes [13].

The C 1s core level spectra reported in Figure 4 (left panels) presents a variety of chemically shifted components. In the pristine delaminated sample, we find six components at 283.92, 284.83, 285.73, 287.0, 288.50 and 289.85 eV BE, associated to the Ti-C-Ti, C-C, C-H_x_, C=O, C-O, ${-CO}_{3}^{2-}$ species, respectively. It is very well noticed that the C atoms are removing electrons from the Ti atoms in MXene at this moment, as indicated by the first peak corresponding to Ti-C-Ti [34], the only peak related to the pure MXene flakes. The other peaks are due to unavoidable carbon contamination, graphitic C-C could be caused by selective Ti dissolution during etching and delamination of Ti_3_C_2_T_x_ sheets. On the other hand, the solvents used in the drying and separation processes, as well as the exposure of the high-surface area material to the ambient atmosphere, are probably responsible in the formation of the C-H_x_ and C-O species [27,35]. A small carbonate contribution is apparent in all C 1s spectra, which could be due to inevitable transfer or handling, and/or to products of electrolyte decomposition [36,37].

As it concerns the O 1s data is shown in Figure 4 (right panels), we observe four components at 530.62, 532.14, 533.48 and 536.47 eV BE. TiO_2_ can be assigned the lowest BE peak, while Ti-C-OH and C=O can be associated with the two next higher-BE peaks, respectively [28,31,38]. The component with the highest B.E corresponds to the -OH functional groups on the surface [39], due to its different electronic environment and also it differs chemically from the Ti–C–OH groups that are bonded within the Ti_3_C_2_T_x_ lattice. These findings make it clear that fitting the O 1s spectra is challenging due to the many overlapping contributions from hydroxides, oxides, and surface terminations. Furthermore, the impurities from other organics and adventitious carbon affected the O 1s spectrum. These impurities are formed by the reactive surface, when stored under air [40,41].

### 3.2. Raman Analysis

Raman spectroscopy is a useful technique for detecting specific bonds and measuring the vibrational energies of functional groups on material surfaces. This study synthesized delaminated MXene (Ti_3_C_2_T_x_) treated with TMAOH. The characteristics of Ti_3_C_2_T_x_, such as flake size, interlayer spacing, surface chemistry, and defect density, are significantly affected by the synthesis and delamination processes [42,43,44]. The lattice vibrations observed in Raman spectra are influenced by these factors, as well as surface terminations (T_x_), intercalated species, and adsorbed species [45]. Hu et al. showed how electrochemical cycling caused changes in the peaks assigned to the =O, −-(OH), and -O (OH) groups. In a recent study, Lioi et al. [46] discovered that the synthesis method has a significant impact on the Raman spectrum of Ti_3_C_2_T_x_ films. This finding indicates that Raman spectroscopy has the potential to serve as a valuable tool for measuring changes in MXene quality based on the synthesis conditions.

The Raman spectra of MXene materials are complicated and contain several regions that correspond to specific vibrational modes, such as the E_g_ mode, which corresponds to in-plane vibrations of surface groups and Ti atoms, and the A_1g_ mode, which is involved with out-of-plane vibrations of the same species. These modes typically appear in the Raman spectrum of pristine Ti-based MXenes within the range of ~200–750 cm^−1^. Additional Raman-active modes such as E_g_ and A_1g_ can also be observed in this region (230–470 cm^−1^ range), in particular the in-plane E_g_ vibration which is affected by surface groups bonded to Ti atoms, making it very responsive to surface chemistry and reactions. Another critical region is the 580–730 cm^−1^ range, which is dominated by C vibrations, including both ‘E_g_’ and ‘A_1g_’ modes. The last region has been utilized for identifying surface groups in different in situ electrochemical studies [47].

**Figure 5:**
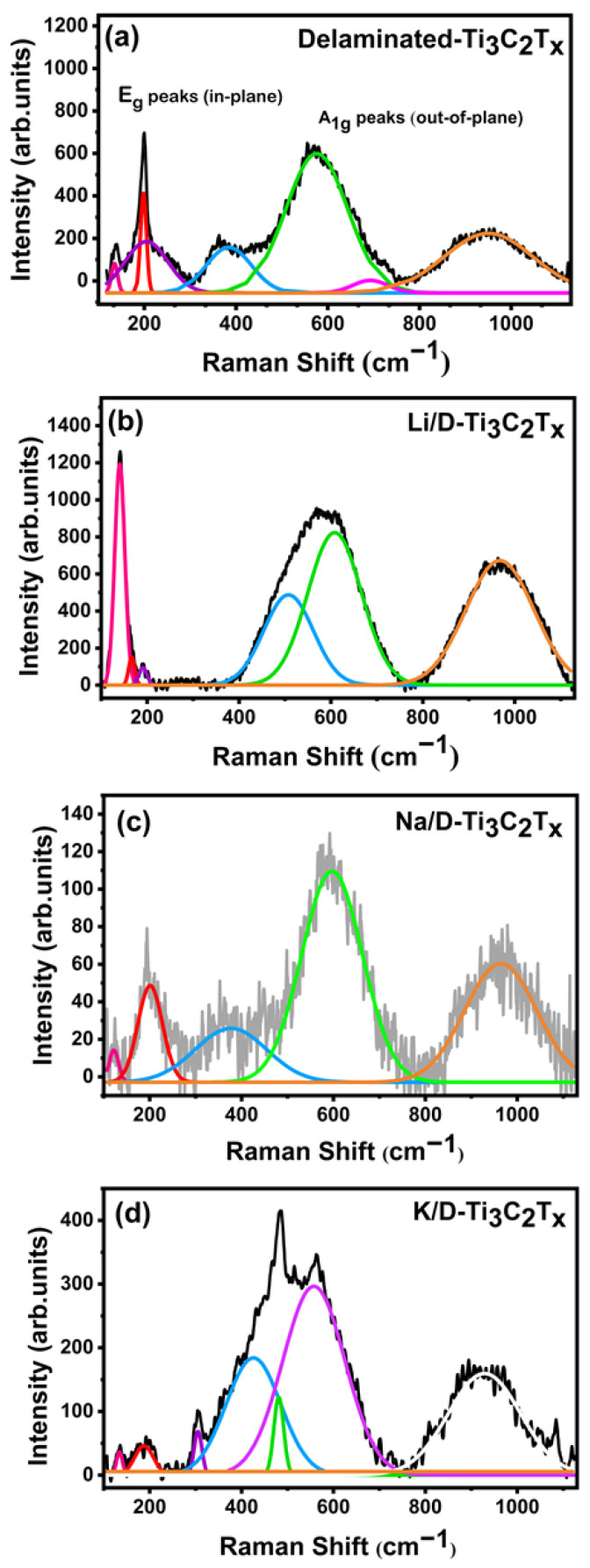
Raman Spectra of (**a**) Pristine Delaminated-MXene (Ti_3_C_2_T_x_); (**b**) Li/D-MXene; (**c**) Na/D-MXene; (**d**) K/D-MXene after 100 cycles of discharge/charge.

Raman analysis was performed for four different samples: the pristine Delaminated-MXene (Ti_3_C_2_T_x_), and those recovered after 100 cycles of discharge/charge with Li, Na and K. All samples display several intense and broad peaks ranging from 100 to 1150 cm^−1^ as shown in Figure 5. The spectra we obtained from pristine delaminated-Ti_3_C_2_T_x_ are smooth compared to the other spectra. After the 100th cycling, the spectra obtained from Li, Na and K measurements are noisy probably because of the passivation layers on the electrodes caused by decomposition of the electrolyte interfere in the Raman analysis. Peak shifting and broadening are caused by surface group distribution and cell distortion. A small band observed at 140 cm^−1^ is the ‘E_g_’ vibrational mode, which may reflect the main contribution from in-plane vibration, and it can be inferred that the in-plane motion of the Ti_2_ and C atoms is weakened by the vibrations of the terminal atoms. This is in accordance with the expansion of the Ti_2_–C bond [48]. Apart from that, there might be possibility of the anatase crystal structure at the same band position (E_g_ vibration mode) due to the in-plane vibrations of Ti_2_ and C atoms [49,50]. In all the spectra, the intense band occurring around 200 cm^−1^ is related with the A_1g_ vibrational mode that is corresponding to the out-of-plane vibration of Ti_2_ and C. Despite strengthening the out-of-plane vibration of C atoms, the terminal group on the surfaces weakens the out-of-plane motion of surface Ti_2_. Furthermore, an intense and broad peak occurring in the 230–470 cm^−1^ region is due to in-plane modes (E_g_) of surface groups (-O, -F, -OH) attached to Ti atoms (as Ti-O, Ti-F or Ti-OH). The 580–730 cm^−1^ region is due to the A_1g_ mode of carbon (C) vibration. There is a sharp intense peak at this A_1g_ (C) region, around 576 cm^−1^ that exhibits the shift to the lower wavenumber in Li, Na and K spectra after cycling. This shifting is observed in the multilayer form, where small interlayer spacing and stacking reduce the vibrations [47,51]. The present observations give significant preliminary insights on the structural and chemical changes of MXene materials during electrochemical cycling, which can impact future operando and depth-resolved studies.

### 3.3. Electrochemical Analysis

**Figure 6:**
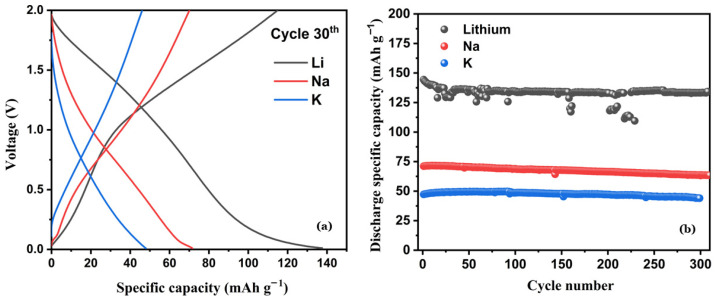
(**a**) Galvanostatic voltage profiles at cycle 30th of D-Ti_3_C_2_T_x_ electrode in alkali metal cells and (**b**) prolonged cycling performance of Li/D-Ti_3_C_2_T_x_ (black line) Na/D-Ti_3_C_2_T_x_ (red line) and K/D-Ti_3_C_2_T_x_ (blue line).

The D-MXene-based electrodes were evaluated in rechargeable half-cell configurations using the different alkali metals (Li, Na and K) as counter electrodes. Cells were assembled in 2032 coin-cell configurations and tested galvanostatically. The cycling current was set at 20 mA/g, and the voltage range was between 0.01 V and 2 V. Each cell was cycled for over 300 cycles to assess the long-term stability of the electrode with different alkali metal anodes. Figure 6a displays the voltage profiles at the 30th cycle for the MXene electrode paired with Li (black), Na (red), and K (blue) metal counter electrodes. The lithium-based cell (black curve) exhibited a distinct discharge plateau between 0.1 V and 0.01 V, which may be attributed to lithium intercalation into the 2D MXene layers and concurrent electrolyte decomposition. Beyond this region, the discharge profile lacks clear plateaus, suggesting a predominance of lithium adsorption in the pores and between the stacked MXene layers. During charging, a short initial plateau is observed, partially reversing the discharge process, followed by a sloped region likely related to the stripping of lithium from the MXene. The voltage profile of the Na-based cell (red curve) is qualitatively similar but shows greater reversibility, indicating more stable electrochemical behavior. In contrast, the K-based cell (blue curve) shows featureless voltage profiles during both discharge and charge, lacking any evident plateaus. This suggests that potassium ions do not intercalate into the MXene structure, possibly due to their larger ionic radius. Figure 6b presents the long-term cycling performance of MXene electrodes with the three alkali metals. All cells demonstrated stable cycling over 300 cycles. The Li-based cell exhibited the highest capacity, ranging from 150 to 125 mAh/g. The Na-based cell delivered a moderate capacity of approximately 75 mAh/g, while the K-based system showed the lowest capacity, around 50 mAh/g roughly one-third of the Li-based cell. This trend correlates with the increasing ionic radius from Li^+^ to K^+^, which limits the extent of ion intercalation and adsorption within the MXene structure.

To clarify the effect of ion size, the diffusion barrier trends observed in the literature were compared with the experimental results. Although the surface diffusion barriers of Li^+^, Na^+^, and K^+^ on MXene surfaces are relatively low [52], they have an additional influence on the rate of ion transport due to ionic radius, interlayer distance, surface terminations (-O, -OH, -F), desolvation energy and formation of SEI [53,54]. The gradual increase in ionic radius (Li^+^, Na^+^, K^+^) progressively limits the possible intercalation reaction in Ti_3_C_2_T_x_. Introduction of Li^+^ gives rise to insignificant lattice distortion and as a result, allows reversible interlayer growth and has development of a well-defined plateau at the greater capacity. The behavior of Na^+^ ion is intermediate, and, on the other hand, the larger K^+^ ion causes major steric hindrances and strains in the lattices, thus preventing bulk intercalation, and promoting surface-based storage [55]. This is in line with the experimentally recorded sloping voltage profiles and reduced capacity. Particularly with electrode fabrication, the partial restacking of MXene sheets that happens inevitably reduces the available gallery height, therefore unfavorably affecting the larger ions. Combinations of Raman peak shift analysis and subsequent cycles XPS surface contributions have proven evidence of increased structural strain and strengthening interfacial reactions in both Na- and K-systems. Overall, the -Li^+^, -K^+^ storage behavior change may be explained by the ionic-radial-related changes in the strain of the lattice, the accessibility of the internal layers, and the interfacial dynamics.

**Figure 7:**
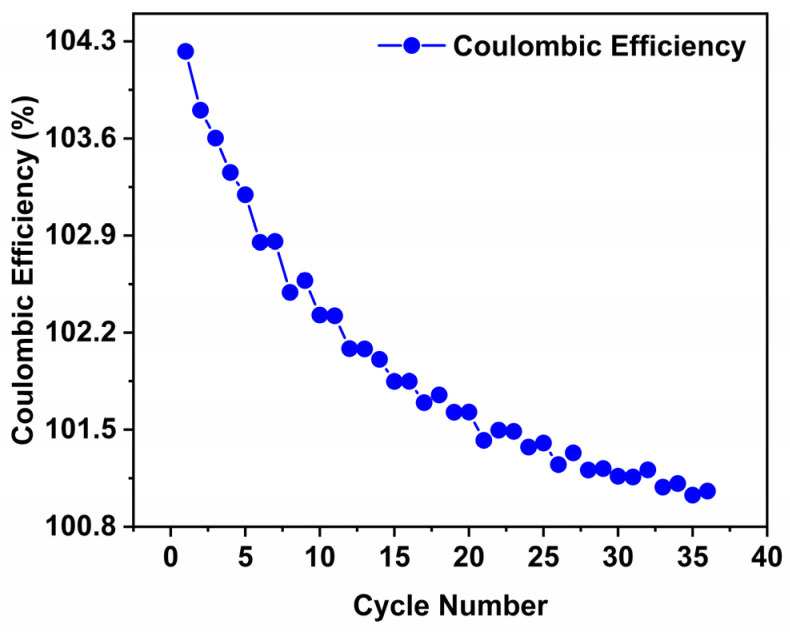
Coulombic efficiency (%) vs. cycle number for Na^+^ cell, highlighting ion-size-dependent reversibility.

To enhance the mechanistic study, the initial Coulombic efficiency (ICE) and cycling Coulombic efficiency (CE) of the Li^+^, Na^+^, and K^+^ cells were measured during cycling. Figure 7 shows the CE of the Na^+^ as a representative example, but the similar behaviour was observed for the other alkali metal (Li^+^ and K^+^) cells.Even though the current study gives priority to comparative mechanistic phenomena under constant conditions as compared to performance metrics optimization, the ICE and CE metrics provide an insight of the irreversible loss of capacity and interfacial phenomena. Li^+^ system has the highest ICE and converges to high-percentage CE, of about 99%, compared to Na^+^ and K^+^ systems which have slightly lower efficiencies, which implies the existence of more irreversible reactions with increasing ionic radius. The results of these observations are consistent with those of the XPS and Raman spectroscopy, thus supporting the ion-size-dependent interfacial evolution in Ti_3_C_2_T_x_ MXene electrodes.

The trends of electrochemical studies are supported by the data collected using X-ray photoelectron spectroscopy (XPS) and Raman spectroscopy. Weakening titanium core-level signals, a gradual development of the O 1s and C 1s spectral components, and the development of alkali-containing species are all indicative of an ion-selective interphase formation. At the same time, lattice strain is indicated by shift and homogeneous broadening of Raman peaks. Such spectroscopic changes are consistent with the changes in a more intercalation-limited Li^+^ storage regime to a regime of combined Na^+^ storage and surface-controlled K^+^ behavior, which is also manifested in the observed differences in capacity and voltage profiles. Due to the ex-situ character of the measurements and the large-scale coverage of the solid electrolyte interface (SEI), strong spectral trends are prioritized in the analysis of the measurements, rather than a quantitative coupling.

## 4. Conclusions

This work investigated behavior of delaminated MXene (Ti_3_C_2_T_x_) based electrodes in rechargeable Li, Na, and K-ion batteries using Raman spectroscopy and XPS and long-term electrochemical testing. Raman spectra analysis showed that there are strong band shifts and broadenings after cycling, which is evidence of quantifiable vibrational changes and structural distortions, which are suggestive of lattice strain and formation of passivation layers. The XPS experiments verified that there was a significant post-cycling surface-chemical evolution of Ti, C, O, and F signatures accompanied by corresponding alkali-metal species (Li, Na, or K), which was therefore in support of the formation of elec-trolyte-formed surface films and SEI- like morphology. Electrochemically, the Li-based cell showed the highest and most consistent performance, maintaining approximately 150–125 mAh g^−1^ over >300 cycles and a clear intercalation relating voltage plateau. The Na-based cell provided an intermediate capacity along with more significantly increased reversibility, and the K-based cell provided the lowest capacity (≈50 mAh g^−1^) and relatively smooth voltage profiles, which is expected to be due to hindered intercalation by the larger ionic radius. These quantitative measurements show conclusively that alkali-metal ion sizes have a strong effect on the charge-store behavior and long-term stability of Ti_3_C_2_T_x_ MXene electrodes in the same conditions. Although the electrochemical trends and ex-situ of spectroscopic results are consistent in terms of ion dependent surface-chemical and structural changes on cycling, they lack direct operando or depth-resolved direct confirmation of the ion penetration or SEI growth processes. With these limitations, delaminated Ti_3_C_2_T_x_, on the one hand, shows significant potential in lithium-ion storage, low efficacy in sodium-ion systems under the current experimental conditions and significant challenges in potassium-ion storage. In turn, this gives strong reasons to conduct further operando and depth-profiling studies as well as surface-engineering and composite-electrode strategies development.

## Figures and Tables

**Table 1:** Parameters obtained for the Li 1s, Na 2s and K 2p Gaussian–Lorentzian curves.

Samples	Region	BE (eV)		FWHM (eV)		Component Atomic %
Lithium/Delaminated-Ti_3_C_2_T_x_	Li1s	54.6		1.0		20%
		55.5		1.7		60%
		56.4		1.7		20%
Sodium/Delaminated-Ti_3_C_2_T_x_	Na2s	63.72		1.8		41%
		64.69		2.2		59%
Potassium/Delaminated-Ti_3_C_2_T_x_	K2p	292.55	295.28	1.3	1.3	60%
		293.26	295.99	1.3	1.2	40%

**Table 2:** Parameters obtained for the C 1s Gaussian–Lorentzian curve fitting of all the samples.

Samples	Region	BE (eV)	FWHM (eV)	Component Atomic %
Delaminated-Ti_3_C_2_T_x_	C1s	283.92	1.3	24%
		284.83	1.4	24%
		285.73	1.5	20%
		287.00	1.8	10%
		288.50	1.8	12%
		289.85	1.7	10%
Lithium/Delaminated-Ti_3_C_2_T_x_	C1s	284.30	1.3	8%
		285.21	1.3	35%
		286.02	1.5	34%
		287.29	1.8	15%
		288.75	1.7	4%
		290.45	1.3	4%
Sodium/Delaminated-Ti_3_C_2_T_x_	C1s	283.73	1.6	3%
		284.92	1.2	12%
		285.69	1.2	30%
		286.54	1.4	32%
		287.66	1.4	17%
		288.78	1.3	6%
Potassium/Delaminated-Ti_3_C_2_T_x_	C1s	283.61	1.0	6%
		284.24	1.0	12%
		284.96	1.2	30%
		285.89	1.6	40%
		287.18	1.2	8%
		288.08	1.2	4%

**Table 3:** Parameters obtained for the O 1s Gaussian–Lorentzian curve fitting all the samples.

Samples	Region	BE (eV)	FWHM (eV)	Component Atomic %
Delaminated-Ti_3_C_2_T_x_	O1s	530.62	1.8	46%
		532.14	1.7	27%
		533.48	1.6	11%
		536.47	2.5	16%
Lithium/Delaminated-Ti_3_C_2_T_x_	O1s	530.85	1.8	20%
		532.33	1.5	40%
		533.25	1.4	20%
		534.40	1.7	20%
Sodium/Delaminated-Ti_3_C_2_T_x_	O1s	531.15	2.0	13%
		532.83	1.7	34%
		533.91	1.6	36%
		535.17	1.7	17%
Potassium/Delaminated-Ti_3_C_2_T_x_	O1s	530.23	1.8	10%
		531.75	1.7	40%
		532.97	1.6	40%
		534.19	2.0	10%

## Data Availability

Data is contained within the article.

## Supplementary Materials

The following supporting information can be downloaded at: https://www.mdpi.com/article/10.3390/nano16050330/s1, Figure S1: XPS survey spectra.

## Abbreviations

The following abbreviations are used in this manuscript:

LIBs	Lithium-ion batteries
SIBs	Sodium-ion batteries
PIBs	Potassium-ion batteries
2D	Two-dimensional
XPS	X-ray photoelectron spectroscopy
DI	De-ionized
TMAOH	Tetramethylammonium hydroxide
PVDF	Polyvinylidene fluoride
NMP	N-Methyl-2-pyrrolidone
UHV	Ultra-high vacuum
BE	Binding Energy
AM	Alkali Metal
SEI	Solid Electrolyte Interface
Ti	Titanium
C	Carbon
O	Oxygen
F	Fluorine
Na	Sodium
Li	Lithium
K	Potassium
