# Supplementary material for: Insight into the Mechanism of MXene Electrodes in Alkali Metal Batteries
Source: Nanomaterials (Basel). 2026 Mar 6;16(5):330. doi: 10.3390/nano16050330 (PMC12986374; doi:10.3390/nano16050330)
Supplement: Supplementary file 1 [file nanomaterials-16-00330-s001.zip › nanomaterials-4158804-supplementary.pdf]

Supplementary Information:

1. X-ray Photoelectron spectroscopy analysis:

XPS survey spectra in a wide BE range are shown in Figure S1, they show the main core level peaks associated with the different elements.

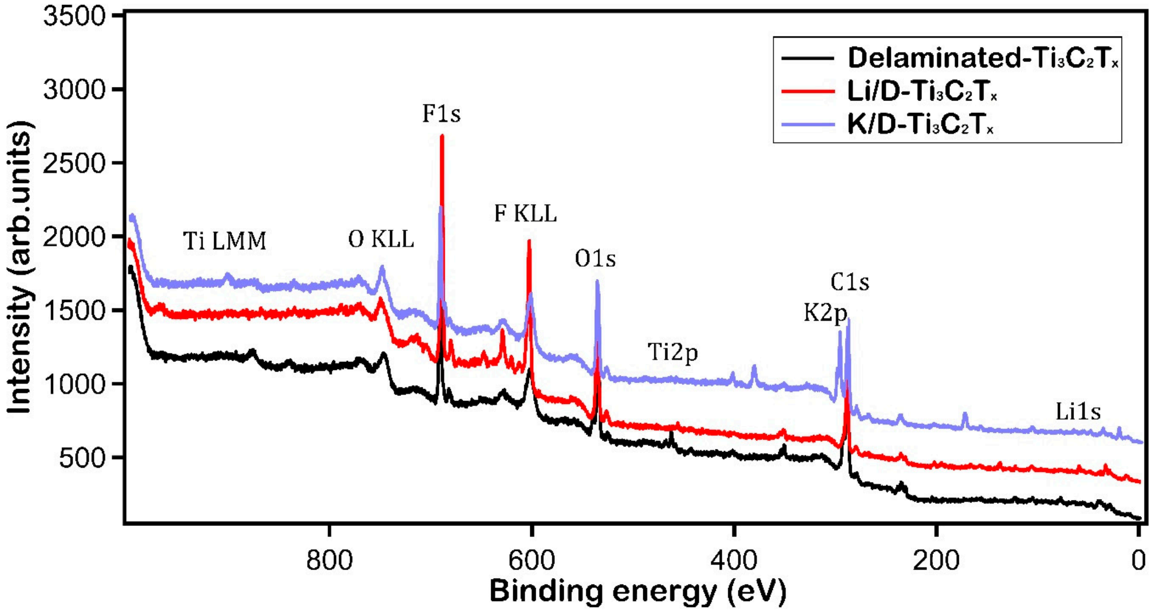

Figure S1. Survey XPS spectra of Delaminated-$Ti_3C_2T_x$ (**Black**); Li-cycled D-$Ti_3C_2T_x$ (**Red**); K-cycled D-$Ti_3C_2T_x$ (**Blue**): spectra are vertically stacked for clarity.
